# Long-term cosmetic outcome after intraoperative radiotherapy boost with low-energy X-rays in breast-conserving therapy: a pooled cohort analysis of the TARGIT-BQR and ROKSM trials

**DOI:** 10.1007/s00066-026-02506-3

**Published:** 2026-02-02

**Authors:** Paulina Schimmelfennig, Christiane Reuter, Uta Kraus-Tiefenbacher, Viktoria Brück, Christina Kaiser, Ralf Keymer, Yasser Abo-Madyan, Katharina Fleckenstein, Benjamin Tuschy, Marc Sütterlin, Frederik Wenz, Hans Reichardt, Mathias Fehr, Markus Kuther, Sylvia Büttner, Elena Sperk

**Affiliations:** 1 Department of Radiation Oncology, University Medical Center Mannheim, Medical Faculty Mannheim, Heidelberg University, Mannheim, Germany; 2 Department of Radiation Oncology, Kantonsspital Münsterlingen, Münsterlingen, Switzerland; 3 Department of Radiation Oncology, Krankenhaus Nordwest, Frankfurt am Main, Germany; 4 Asklepios Klinik Lich GmbH, Lich, Germany; 5 University Medical Center Bonn, Medical Faculty Bonn, Bonn University, Bonn, Germany; 6 Klinikum Kassel, Kassel, Germany; 7 Department of Gynecology and Obstetrics, University Medical Center Mannheim, Medical Faculty Mannheim, Heidelberg University, Mannheim, Germany; 8 Medical Faculty, University of Freiburg, University Hospital Freiburg, Freiburg, Germany; 9 Department of Gynecology and Obstetrics, Kantonsspital Frauenfeld, Frauenfeld, Switzerland; 10 Department of Gynecology and Obstetrics, Kantonsspital Münsterlingen, Münsterlingen, Switzerland; 11 Department of Medical Biometry, University Medical Center Mannheim, Medical Faculty Mannheim, Heidelberg University, 68167 Mannheim, Germany; 12 Mannheim Cancer Center, University Medical Center Mannheim, Medical Faculty Mannheim, Heidelberg University, Mannheim, Germany

**Keywords:** Breast cancer, IORT, Boost, Cosmetic outcome, Comesis

## Abstract

**Purpose:**

This study investigates objective long-term cosmetic outcomes in patients with breast cancer after intraoperative radiotherapy applied as a boost (IORT boost) from a pooled international cohort.

**Methods:**

A pooled analysis of two prospective studies with IORT boost (low-energy X‑rays, 20 Gy; inclusion criteria: 3.5 cm maximum tumor size and preoperative indication for a boost according to local criteria) during breast-conserving surgery followed by whole-breast radiotherapy (46–50 Gy). The analysis included photos from a subgroup of the prospective phase IV TARGeted Intraoperative radioTherapy (TARGIT) Boost Quality Registry (BQR) study (NCT01440010) with a follow-up of up to 10 years as well as patients from a Swiss study (ROKSM; Spital Thurgau AG; NCT02114086) with a follow-up of 5 years. The pooled analysis included photos from both trials taken at the same timepoints (6 weeks, 6 months, 1 year, 2 years, and 5 years after completion of whole-breast radiotherapy). Cosmetic results were evaluated with the validated BCCT.core software (BCCT.core 2.0, INESC Porto, Portugal) to assess symmetry, color, and scars. A generalized estimating equation (GEE) regression model was used to compare the two cohorts over time.

**Results:**

The pooled analysis included 777 cosmetic assessments from 276 patients. Across all follow-ups in the pooled analysis, the majority of cosmetic results were rated as excellent or good (61.9%). Patients from TARGIT-BQR had significantly more excellent or good ratings than patients from ROKSM (*p* < 0.0001). The proportion of patients receiving “excellent” and “good” ratings remained higher in TARGIT-BQR than in ROKSM at every timepoint during follow-up. Overall, for up to 10 years, the majority of ratings in the whole cohort were “excellent” or “good.”

**Conclusion:**

In this international pooled analysis, the cosmetic outcome after IORT boost followed by whole-breast irradiation shows a good esthetic long-term outcome. Further studies should explore factors that influence cosmetic outcomes and consider additional treatment-related parameters.

**Supplementary Information:**

The online version of this article (10.1007/s00066-026-02506-3) contains supplementary material, which is available to authorized users.

## Introduction

Breast cancer is the most common malignancy among women in industrialized countries and is one of the most frequently irradiated cancers. Randomized studies have demonstrated the equivalence of breast-conserving therapy (BCT) followed by external-beam radiotherapy (EBRT) compared to radical mastectomy in terms of overall survival in breast cancer patients [1–3]. Since most local recurrences appear in close proximity to the tumor bed, a boost to the tumor bed can further decrease local recurrences in addition to whole-breast radiotherapy (WBRT) [4–6] and is therefore considered standard in the national guideline of the German-speaking region for patients with certain risk factors [7]. Various methods are used for boost application to the breast, with no particular application technique in favor [8]. With intraoperative radiotherapy (IORT) as a boost technique using low-energy X‑rays or electrons, a highly effective dose is delivered directly to the tumor bed during surgery, while minimizing exposure to surrounding healthy tissue. This targeted application maximizes biological effectiveness, ensuring optimal local control [9]. The advantages of intraoperative radiotherapy include high precision of beam application (reduction of geographical miss) directly to the tumor bed during surgery; the possibility of sparing the skin; and preventing tumor cell proliferation between surgery and the start of any adjuvant radio- or chemotherapy, so called temporal miss [9, 10]. Compared to conventional external boost techniques, IORT can shorten the overall treatment time and therefore reduce the number of visits to the radiotherapy department.

With improvement of breast cancer survival [11], esthetic outcome has gained increasing importance. Especially in the context of shared decision-making, secondary outcomes are an important consideration when evaluating different treatment options. Radiotherapy can lead to notable changes in color, for example hyperpigmentation of the breast or telangiectasia. Additionally, fibrosis resulting from either surgery or radiotherapy or a combination of both can affect symmetry [12]. Given that IORT boost is a highly localized radiation technique, the addition of whole-breast radiotherapy may compromise the overall cosmetic outcome due to cumulative tissue effects. This concern is supported by large boost studies, which have shown that adding a boost to whole-breast irradiation is associated with poorer cosmetic results [13]. Additionally different radiotherapy techniques have different effects on cosmetic outcome [14–21]. To date, most studies on IORT boost have focused on local recurrence and acute or chronic side effects, while there are few analyses of cosmetic outcome. A subgroup of the TARGIT-BQR cohort (NCT01440010) has already been analyzed in detail regarding cosmetic outcomes as assessed by three different experts using the Harvard scale and published by Goerdt et al. [22]. The current analysis used BCCT.core (BCCT.core 2.0, INESC Porto, Portugal), an objective tool that enables standardized and reproducible assessment of cosmetic outcomes instead of individual ratings by physicians. To validate the data from TARGIT-BQR in another prospective cohort, we performed a pooled analysis of available TARGIT-BQR patients and those from a prospective study from Switzerland (Swiss ROKSM study, NCT02114086) with long-term data after an IORT boost with low-energy X‑rays and compared the results.

## Materials and methods

### Cohort

Both registry studies included patients with breast cancer who had a clinical indication to receive a boost in addition to whole-breast irradiation according to local criteria which were based on national guidelines [7]. Study-specific inclusion criteria for both studies were a tumor size ≤ 3.5 cm (T1–2) and written informed consent. Exclusion criteria were lack of consent, tumor size > 3.5 cm, or lack of cooperation/no preoperative indication for a boost.

The ROKSM study of the Swiss hospital Spital Thurgau AG started in December 2012 and ended in January 2020, with a follow-up of 5 years (registered on clinicaltrials.gov under the identifier NCT02114086). In total, 85 patients with 86 cases (one patient with bilateral breast cancer) were included, and photos from 83 patients and cases were available for the current analysis.

The TARGIT-BQR study started in September 2011 and ended in December 2020, including over 1000 patients from 10 different German centers with a maximum follow-up of 10 years (registered on clinicaltrials.gov under the identifier NCT01440010). Cosmetic data in TARGIT-BQR were collected exclusively at the Mannheim University Hospital center from 219 patients with 220 cases.

All patients from both studies gave informed consent, and both studies had approval from an ethics committee.

### Treatment

Following surgical removal of the tumor during breast-conserving surgery, the intraoperative boost radiation was delivered to the tumor bed directly using the INTRABEAM® low-energy X‑ray system (Carl Zeiss Meditec AG, Oberkochen, Germany). In both cohorts, patients received either 20 Gy or 12 Gy, with the lower dose applied when the skin-to-applicator distance was less than 1 cm. The majority received 20 Gy. Median irradiation time was 28 min (11–53 min) for the TARGIT-BQR cohort and 17 min (6–50 min) for the ROKSM study. Applicator sizes ranged from 30 to 50 mm in the TARGIT-BQR cohort and from 20 to 50 mm in the ROKSM cohort, with a median of 40 mm for TARGIT-BQR and 35 mm for ROKSM. Afterwards, all patients received whole-breast irradiation. Whole-breast irradiation was administered following complete wound healing and/or chemotherapy. An interval of at least 5–6 weeks between surgery and irradiation was maintained, as shorter intervals are associated with a higher risk of side effects [23]. A standard total dose of 46 Gy was applied in 2‑Gy fractions, or 50 Gy in 2‑Gy fractions in cases with additional risk factors like positive margins without re-resection or positive lymph nodes.

### Follow-up

In the ROKSM study, patients were followed up prospectively after completed WBRT at 6 weeks, 6 months, 1 year, 2 years, and 5 years. In the TARGIT-BQR study, patients also received a follow-up at 6 weeks and 6 months after WBRT, from then on yearly up to 10 years. Cosmetic data were collected at all these follow-ups as well as prior to IORT in the ROKSM study and after IORT but before starting WBRT in the TARGIT-BQR study. Although follow-up continued for 10 years in the TARGIT-BQR study, no cosmetic photographs were available at the 10-year timepoint, with the last images taken at year 9. Cosmetic data were collected in a standardized procedure with photos of the breast area in three different positions (arms up, arms down, and from the treated side). In this analysis, only the photos from the front with arms down were used. Table 1 shows the number of evaluated photos for each cohort and follow-up. Photo documentation was not consistently available for all patients at every timepoint, so the photos per timepoint may vary. Photos of insufficient quality (e.g., out of focus, both breasts not fully visible, poor lighting) were excluded from the analysis.

**Table 1:** Overview of all follow-ups and the available number of photo documentations for each cohort

Follow-ups with photos	All patients from both cohorts	TARGIT-BQR cohort	ROKSM cohort
Before IORT	74	–	74
After IORT and before WBRT	163	163	–
After 6 weeks	161	101	60
After 6 months	161	85	76
After 1 year	176	106	70
After 2 years	168	110	58
After 3 years	76	76	–
After 4 years	75	75	–
After 5 years	111	56	55
After 6 years	37	37	–
After 7 years	21	21	–
After 8 years	7	7	–
After 9 years	3	3	–
After 10 years	0	0	–

*WBRT *whole-breast radiation therapy

### Cosmetic assessment

Only photos with frontal views of the breast area were used in the analysis with the BCCT.core software. BCCT.core is an objective, user-independent tool to evaluate cosmetic outcome after breast-conserving surgery. With reference points at nipples, breast contour, and the sternal notch, the software evaluates asymmetry, color changes, and scars. The results are translated into categories of the Harrison scale: excellent (treated breast is almost identical to the untreated breast), good (treated breast shows slight dissimilarities compared to the untreated breast), fair (treated breast is different to the untreated breast but not distorted), poor (treated breast is clearly distorted compared to the untreated breast) [24, 25]. Examples of the BCCT.core evaluation are shown in Supplement figure 1. All patients gave informed consent to the anonymous publication of the photos.

### Statistical analysis

The results of both studies were analyzed individually for each of their follow-ups. The pooled analysis only considered the joint follow-ups at 6 weeks, 6 months, 1 year, 2 years, and 5 years. Differences between the cohorts were compared using the chi-square test, t‑test, or Mann–Whitney U test. A generalized estimating equation (GEE) regression model was used to compare the two cohorts over time. For cosmetic outcome, neither a predefined statistical hypothesis nor adjustment for multiple testing was performed.

All statistical calculations were performed using the SAS software, release 9.4 (SAS Institute, Inc, Cary, NC, USA). A *p*-value of less than 0.05 was considered significant.

## Results

### Patient characteristics

The mean age at the time of IORT was 59 ± 10.5 years in TARGIT-BQR and 60.3 ± 9.8 years in the ROKSM cohort. All systemic treatments were applied following the local interdisciplinary tumor board and guideline recommendations. Table 2 presents key patient characteristics, detailed tumor characteristics, and treatment details.

**Table 2:** Patient, tumor, and treatment characteristics

General		Value		
		Combined cohort *n* = 303 breast cancers	TARGIT-BQR cohort *n* = 219 with 220 breast cancers	ROKSM cohort *n* = 83
Location	Left breast	160 (52.8)	117 (53.2)	43 (51.8)
	Right breast	143 (47.2)	103 (46.8)	40 (48.2)
	Upper outer quadrant	144 (47.8)	107 (48.6)	37 (45.7)
	Upper inner quadrant	56 (18.6)	48 (21.8)	8 (9.9)
	Lower outer quadrant	35 (11.6)	26 (11.8)	9 (11.1)
	Lower inner quadrant	13 (4.3)	10 (4.6)	3 (3.7)
	Center of both upper quadrants	19 (6.3)	12 (5.5)	7(8.6)
	Center of both lower quadrants	7 (2.3)	2 (0.9)	5 (6.2)
	Central	11 (3.7)	7 (3.2)	4 (4.9)
	Center of both outer quadrants	12 (4.0)	5 (2.3)	7 (8.6)
	Center of both inner quadrants	4 (1.3)	3 (1.4)	1 (1.23)
	Not specified	2	–	2
Family history for breast cancer	Positive	93 (30.9)	83 (37.9)	10 (12.2)
	Negative	208 (69.1)	136 (62.1)	72 (87.8)
	Not specified	2	1	1
Tumor size	Median diameter (cm)	1.5	1.5	1.8
T	0	1 (0.3)	1 (0.5)	–
	1	212 (71.6)	159 (74.3)	53 (64.6)
	2	82 (27.7)	53 (24.8)	29 (35.4)
	3	1 (0.3)	1 (0.5)	–
	Not specified	7	6	1
N	0	235 (78.1)	173 (79.0)	62 (75.6)
	1	58 (19.3)	39 (17.8)	19 (23.2)
	2	4 (1.3)	4 (1.8)	–
	3	4 (1.3)	3 (1.4)	1 (1.2)
	Not specified	2	1	1
M	0	254 (98.5)	188 (98.9)	66 (97.1)
	1	4 (1.6)	2 (1.1)	2 (2.9)
	Not specified	45	30	15
L	0	211 (73.8)	160 (76.9)	51 (65.4)
	1	75 (26.2)	48 (23.1)	27 (34.6)
	Not specified	17	12	5
V	0	276 (97.2)	204 (98.6)	72 (93.5)
	1	8 (2.8)	3 (1.4)	5 (6.5)
	Not specified	19	13	6
G	1	54 (18.4)	42 (19.1)	12 (14.6)
	2	163 (55.6)	115 (54.5)	48 (58.5)
	3	76 (25.9)	54 (25.6)	22 (26.8)
	Not specified	10	9	1
R	0	281 (95.6)	205 (95.8)	76 (95.0)
	1	13 (4.4)	9 (4.2)	4 (5.0)
	Not specified	9	6	3
Estrogen receptor	Positive	259 (87.8)	185 (86.8)	74 (90.2)
	Negative	36 (12.2)	28 (13.21)	8 (9.8)
	Not specified	8	7	1
Progesterone receptor	Positive	240 (81.9)	176 (83.4)	64 (78.1)
	Negative	53 (18.1)	35 (16.6)	18 (21.9)
	Not specified	10	9	1
HER2neu	Positive	43 (14.3)	37 (17.0)	6 (7.3)
	Negative	257 (85.7)	181 (83.0)	76 (92.7)
	Not specified	3	2	1
Chemotherapy	Yes	111 (36.9)	85 (39.0)	26 (31.3)
	No	190 (63.1)	133 (61.0)	57 (68.7)
	Not specified	2	2	–
Endocrine therapy	Yes	190 (63.1)	116 (53.2)	74 (89.2)
	No	111 (36.9)	102 (46.8)	9 (10.8)
	Tamoxifen	142 (47.3)	93 (42.7)	49 (59.8)
	Aromatase inhibitors	81 (26.9)	22 (10.1)	59 (71.1)
	Tamoxifen + GnRH analogue	5 (1.7)	3 (1.4)	2 (2.4)
Trastuzumab	Yes	30 (10.0)	24 (11.0)	6 (7.2)
	No	271 (90.0)	194 (89.0)	77 (92.8)
	Not specified	2	2	–
Re-resection	Yes	86 (29.9)	34 (16.0)	52 (68.4)
	No	202 (70.1)	178 (84.0)	24 (31.6)
	Not specified	15	8	7
IORT dose	Median	20	20	20
WBRT total dose	Range (median)	46–50*	46–50 (50)	46–50*

Data are shown for the combined cohort as well as separately for each cohort; data are shown as *n* (%) or median; due to rounding, there may be slight deviations from 100%

*IORT* intraoperative radiation therapy, *WBRT* whole-breast radiation therapy, *GnRH *gonadotropin-releasing hormone

*The distinction between 46 and 50 Gy for WBRT was not available for the ROKSM cohort

### Cosmetic outcome

**Fig. 1:**
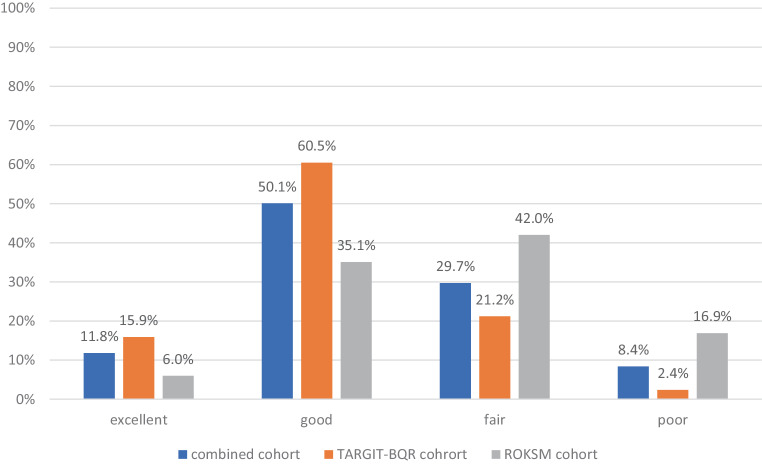
Overall cosmetic analysis over 5 years of follow-up for the combined as well as for each individual cohort; data shown as *n* (%); due to rounding, there may be slight deviations from 100%

**Fig. 2:**
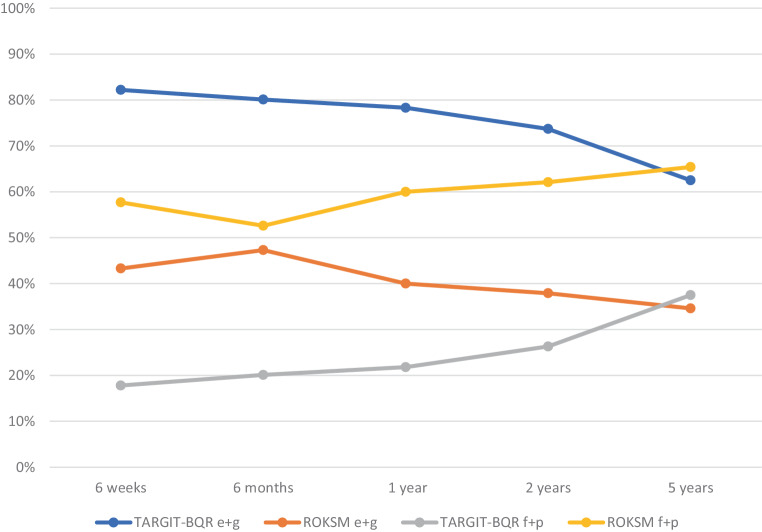
Excellent and good (*e* *+* *g*) vs. fair and poor (*f* *+* *p*) cosmetic outcomes over time for the common follow-ups of both cohorts in relation to the available patients at the certain timepoints.

In the pooled evaluation of both studies, the majority of cosmetic results after 5 years were rated as “excellent” or “good” (61.9%; Fig. 1). In the TARGIT-BQR cohort, a majority of excellent and good ratings were observed at every timepoint, even beyond 5 years. When comparing the individual cosmetic results of the identical follow-up timepoints at 6 weeks, 6 months, 1 year, 2 years, and 5 years of both cohorts using the chi-square test, a significant difference is observed at all time points: the proportion of patients with the ratings “poor” and “fair” is higher in the ROKSM cohort at all timepoints compared to the TARGIT-BQR cohort (Table 3; Fig. 2).

**Table 3:** Cosmetic analysis for each timepoint and cohort

Follow-up timepoints		BCCT.core^a^ evaluation				Chi-square test
		Excellent	Good	Fair	Poor	
Before IORT	TARGIT-BQR cohort	–	–	–	–	–
	ROKSM cohort	21 (28.4)	48 (64.9)	5 (6.8)	0 (0.0)	
After IORT before WBRT	TARGIT-BQR cohort	35 (21.5)	101 (62.0)	27 (16.5)	0 (0.0)	–
	ROKSM cohort	–	–	–	–	
After 6 weeks	TARGIT-BQR cohort	20 (19.8)	63 (62.4)	17 (16.8)	1 (1.0)	*p* < 0.001
	ROKSM cohort	2 (3.3)	24 (40.0)	25 (41.7)	9 (15.0)	
After 6 months	TARGIT-BQR cohort	15 (17.6)	53 (62.4)	15 (17.6)	2 (2.4)	*p* < 0.001
	ROKSM cohort	2 (2.6)	34 (44.7)	32 (42.1)	8 (10.5)	
After 1 year	TARGIT-BQR cohort	12 (11.3)	71 (67.0)	20 (18.9)	3 (2.8)	*p* < 0.001
	ROKSM cohort	6 (8.6)	22 (31.4)	30 (42.9)	12 (17.1)	
After 2 years	TARGIT-BQR cohort	19 (17.3)	62 (56.4)	26 (23.6)	3 (2.7)	*p* < 0.001
	ROKSM cohort	5 (8.6)	17 (29.3)	23 (39.7)	13 (22.4)	
After 3 years	TARGIT-BQR cohort	14 (18.4)	40 (52.6)	19 (25.0)	3 (4.0)	–
	ROKSM cohort	–	–	–	–	
After 4 years	TARGIT-BQR cohort	11 (14.7)	43 (57.3)	19 (25.3)	2 (2.7)	–
	ROKSM cohort	–	–	–	–	
After 5 years	TARGIT-BQR cohort	7 (12.5)	28 (50.0)	19 (33.9)	2 (3.6)	*p* = 0.0059
	ROKSM cohort	4 (7.3)	15 (27.3)	24 (43.6)	12 (21.8)	
After 6 years	TARGIT-BQR cohort	4 (10.8)	23 (62.2)	10 (27.0)	0 (0.0)	–
	ROKSM cohort	–	–	–	–	
After 7 years	TARGIT-BQR cohort	3 (14.3)	10 (47.6)	8 (38.1)	0 (0.0)	–
	ROKSM cohort	–	–	–	–	
After 8 years	TARGIT-BQR cohort	1 (14.3)	3 (42.9)	3 (42.9)	0 (0.0)	–
	ROKSM cohort	–	–	–	–	
After 9 years	TARGIT-BQR cohort	0 (0.0)	3 (100.0)	0 (0.0)	0 (0.0)	–
	ROKSM cohort	–	–	–	–	
After 10 years	TARGIT-BQR cohort	–	–	–	–	–
	ROKSM cohort	–	–	–	–	

Data are shown as *n* (%), *p*-value; due to rounding, there may be slight deviations from 100%

*IORT* intraoperative radiation therapy, *WBRT* whole-breast radiation therapy

^a^BCCT.core 2.0, INESC Porto, Portugal

In a further analysis, we analyzed whether the BCCT.core results differed over time between the two cohorts. In the GEE computational model, the BCCT.core results of the two groups were considered at the comparable 5 follow-up timepoints (6 weeks, 6 months, 1 year, 2 years, 5 years). In the model, 777 datasets, i.e., cosmetic results with BCCT.core, from 276 patients of both cohorts could be combined.

When comparing the cosmetic results across all shared timepoints of the analysis and evaluating the two cohorts based on the BCCT.core values, a significant difference is observed for the “cohort” effect (*p* < 0.0001 in the chi-square test). In the TARGIT-BQR cohort, the proportion of patients with ratings of “good” and “excellent” is higher compared to the ROKSM study. This confirms the differences already highlighted between the cohorts across all timepoints (Table 3).

When both cohorts are considered together over time, a significant effect of “time” is observed, with a *p*-value of 0.0227 in the chi-square test. For this analysis, the timepoint 6 weeks was compared to those at 6 months, 1 year, 2 years, and 5 years. The difference in cosmetic results emerged when comparing outcomes at 6 weeks or 6 months (*p* = 0.0439 in the chi-square test) and 5 years (*p* = 0.0155 in the chi-square test): ratings of “good” and “excellent” outcomes declined, while ratings of “fair” and “poor” outcomes increased. No other time-based comparisons showed significant differences (Table 4).

**Table 4:** Cosmetic analysis at the timepoints 6 weeks and 5 years for the combined cohort

BCCT.core^a^ evaluation	Timepoint		
	6 weeks	6 months	5 years
Excellent	22 (8.1)	17 (6.3)	11 (4.0)
Good	87 (32.0)	87 (32.0)	43 (15.8)
Fair	42 (15.4)	47 (17.3)	43 (15.8)
Poor	10 (3.7)	10 (3.7)	14 (5.2)

Data are shown as *n* (%); due to rounding, there may be slight deviations from 100%

^a^BCCT.core 2.0, INESC Porto, Portugal

However, when considering the effect of “cohort and time” in the comparative analysis, i.e., the cosmetic results over time separated by the “TARGIT-BQR cohort” and “ROKSM cohort,” no interaction between the groups was observed in the BCCT.core results (*p* = 0.7287 in the chi-square test). The proportion of patients with “poor” and “fair” ratings remained higher in the ROKSM study at all timepoints compared to the BQR study. Conversely, the proportion of “good” and “excellent” ratings was higher in the TARGIT-BQR study.

We analyzed several parameters, including radiation dose, irradiation time, applicator size, patient age, and tumor size, to find a possible explanation for the differences in cosmetic outcome. Further analysis revealed significant differences between the cohorts: the ROKSM cohort had larger tumor sizes (*p* = 0.0011 in Mann–Whitney U test), while the TARGIT-BQR cohort was characterized by the use of larger applicators (*p* < 0.001 in Mann–Whitney U test) and longer irradiation times (*p* < 0.001 in Mann–Whitney U test).

## Discussion

Most IORT studies focus on oncological outcomes, particularly local recurrence rates, while results for long-term toxicities are less common. Although the cosmetic outcome after breast cancer treatment is a key factor for quality of life [26, 27] and an important parameter in shared decision-making, data on cosmetic outcomes remain limited for breast cancer patients with a low-energy X‑ray IORT boost. Specifically, objective assessment of cosmetic results is challenging. To date, there is currently no standardized objective tool available to evaluate the cosmetic outcomes after breast-conserving treatment. Traditional approaches to assess the cosmetic outcomes of breast-conserving surgery often rely on subjective evaluations by patients themselves or examination of photos by one or more experts, as do recently published and ongoing trials [22, 28–30].

Various methods have been employed to assess cosmetic outcomes. Harris et al. (1979) introduced a subjective scoring system categorizing outcomes as excellent, good, fair, or poor [24]. First attempts at objective evaluation after breast surgery were made shortly after, with the Breast Retraction Assessment (BRA) by Pezner et al. [31]. Cardoso et al. furthered this approach by creating a software (BCCT.core) utilizing digital images to evaluate cosmetic outcomes of breast-conserving treatment. BCCT.core combines objective measures of asymmetry, skin color, and scarring to generate an overall cosmetic score based on Harris et al.’s classification system [25]. The validated software therefore enables reproducible, objective evaluation of esthetic outcomes in breast cancer conservative treatment using digital photos [32]. We chose an objective tool of cosmetic evaluation in order to minimize any kind of bias that may come with subjective evaluation. Although one may argue that a patient’s subjective assessment is the most important, an objective analysis of this often-overlooked secondary outcome is crucial for establishing a standardized basis of comparison. A recent study by Corica et al. compared four different cosmetic rating scales, subjective as well as objective, and showed little agreement between them. Especially the patient’s evaluation did not always correlate with the others. They therefore recommend using a combination of subjective and objective evaluations in future analyses [33].

Our evaluation with BCCT.core shows IORT boost to be a procedure with satisfying long-term cosmetic outcomes. The pooled analysis consistently showed a majority of good to excellent cosmetic outcomes at all timepoints, indicating that the use of an IORT boost does not negatively impact cosmetic outcomes. While the separate analysis of each cohort revealed differences in cosmetic outcomes, which are discussed in detail later, the cosmetic results remained consistently stable over time for each group. Smaller subjective assessments [22, 28–30] as well as recent studies that analyzed cosmetic outcomes with BCCT.core after IORT as a single high dose in the TARGIT‑A trial [33, 34] provide additional evidence for a good cosmetic outcome. Intraoperative radiation therapy as a single-shot radiotherapy without WBRT has been shown to result in better cosmetic outcomes than standard WBRT [35]. Consequently, several studies have also demonstrated superior cosmetic results with IORT alone compared to IORT followed by WBRT [30, 34]. One contributing factor is the discoloration from hyperpigmentation and telangiectasia, which is linked to the higher doses and larger treatment volumes used in WBRT [12, 34]. Wang et al. compared two recent boost techniques: simultaneous integrated tumor bed boost (SIB) and sequential boost during hypofractionated whole-breast irradiation after breast-conserving surgery: SIB outcomes were significantly better compared to sequential boost, with low deterioration rates after radiation for both techniques (9.8% vs. 7.6%; *p* = 0.22) [36]. Deterioration rates were 17% in the TARGIT-BQR cohort and 12% in the ROKSM cohort (6 months vs. 5 years).

Specifically, Goerdt et al. investigated long-term cosmetic outcomes after IORT boost followed by WBRT using the same cohort from the University Hospital Mannheim as in the TARGIT-BQR study. However, in contrast to this study, they conducted a cosmetic analysis by evaluating photos taken from three different positions, assessed by three different experts and not by using the BCCT.core software. The study also had a shorter follow-up period of only 4 years, with slightly different follow-up timepoints. Similar to the findings in this study, the majority of results for each timepoint were rated as good or excellent [22]. Our study can confirm these findings through a different, objective assessment method, also extending the long-term results by several years and providing a comparison with an additional cohort.

Further evidence on alternative IORT boost techniques also supports favorable cosmetic outcomes. Studies investigating intraoperative electron radiotherapy (IOERT) followed by whole-breast irradiation consistently reported good to excellent results, in some cases superior to those achieved with external-beam boosts, with low rates of high-grade toxicity [37–39]. These findings support our results with low-energy X‑ray IORT and underscore the potential of IORT boosts as an esthetics-preserving treatment option.

While both cohorts underwent the same radiotherapeutic regimen (BCS with IORT boost followed by WBRT), we observed significant differences in cosmetic outcomes between them. A comparison of both studies suggests that despite following the same therapeutic steps, cosmetic outcomes are likely influenced by numerous additional factors. In this comparison, the ROKSM study consistently reported poorer cosmetic outcomes. Several factors may account for this disparity. The ROKSM cohort demonstrated significantly more continuous and thorough photo documentation for each patient compared to the subgroup of the TARGIT-BQR study. Since the TARGIT-BQR study is a so-called no-budget registry study (meaning it operates without funding, with all services provided purely out of academic interest), it is possible that the less-structured invitation and photography process led to fewer documented cosmetic outcomes overall. However, numerous factors influence cosmetic outcomes, including radiotherapy, patient factors, tumor characteristics, and surgical technique. Both studies offered oncoplastic procedures for breast reconstruction, yet it is unclear from the registry data whether these were utilized. The ROKSM study involved two different surgical sites with two distinct surgical teams, offering a potential explanation. Additionally, the literature suggests that while oncoplastic surgeries may improve cosmetic outcome [40, 41], there is an imprecise definition of the term, as these techniques are individualized based on breast characteristics [12, 42]. While patient age was evenly distributed in both studies, the tumor size was significantly different between them, and more re-excisions occurred in the ROKSM study. Larger tumor size is a well-established risk factor for higher re-excision rates, as achieving clear margins becomes technically more challenging and microscopic extensions may be more common. This potentially indicates larger resected volumes, which could lead to poorer cosmetic outcomes. Surprisingly, the TARGIT-BQR study achieved better cosmetic outcomes, despite employing significantly larger applicators and longer irradiation times—parameters usually linked to poorer results due to greater tissue impact. This unexpected finding underscores the considerable influence of other factors. Our results show that surgical technique and initial tumor size might be crucial determinants of cosmetic outcome, with larger tumors and more frequent re-operations in the ROKSM study likely causing more pronounced tissue defects that counteracted the potential negative effects of the radiotherapy parameters in the TARGIT-BQR group.

Our findings indicate that while radiotherapy and its techniques might play an important role in shaping cosmetic outcomes, they are possibly not the sole determinants. Surgical technique, photo documentation quality, and patient-specific characteristics might also be considerable factors. This underscores the need for future prospective studies to systematically evaluate these variables.

## Limitations

The combined analysis of both studies provided a substantial number of long-term cosmetic outcomes. Unfortunately, the follow-up intervals in the two studies were not fully aligned, resulting in some data loss in the joint analysis. To improve data quality in future analyses, larger multicenter studies would be beneficial.

The BCCT.core analysis software offers an objective and consistent method for evaluating cosmetic results. However, only frontal images were used in this analysis, meaning that potentially valuable information from images in other positions was lost. Expanding this tool to include additional image perspectives in the future could improve accuracy further. Additionally, subjective assessments by patients themselves were not included in this study.

The cohorts had some significantly different treatment and patient characteristics that may have contributed to the differences observed between them. In particular, the lack of information on the use of oncoplastic breast surgery could have played an important role. It is also challenging to attribute cosmetic outcomes solely to radiotherapy. As noted in the discussion, various aspects of treatment and patient characteristics play an important role. None of these influencing factors were considered in this study, though including them in future analyses would be highly valuable.

## Conclusion

In this objective assessment, IORT as a boost appears to result in good long-term cosmetic outcomes for a variety of patients. Most patients had a good or excellent outcome, with a decline through the years. It is recommended to consistently include cosmetic outcomes along with their relevant influencing parameters in future studies.

## Supplementary Information

ESM1: Supplementary material 1
