# Supplementary material for: Long-term cosmetic outcome after intraoperative radiotherapy boost with low-energy X-rays in breast-conserving therapy: a pooled cohort analysis of the TARGIT-BQR and ROKSM trials
Source: Strahlenther Onkol. 2026 Feb 2;202(7):785–95. doi: 10.1007/s00066-026-02506-3 (PMC13290794; doi:10.1007/s00066-026-02506-3)
Supplement: Supplementary file 1 — ESM1: Supplementary material 1 [file 66_2026_2506_MOESM1_ESM.docx]

Online Supplement

Article title: “Long-term cosmetic outcome after intraoperative radiotherapy boost with low kV X-rays in breast-conserving therapy: A pooled cohort analysis of the TARGIT-BQR and ROKSM trial”

Journal name: Strahlentherapie und Onkologie

# Index of Supplementary Figures and Tables

Figure S1: Examples for BCCT.core categories

# Figure S1: Examples for BCCT.core categories

| 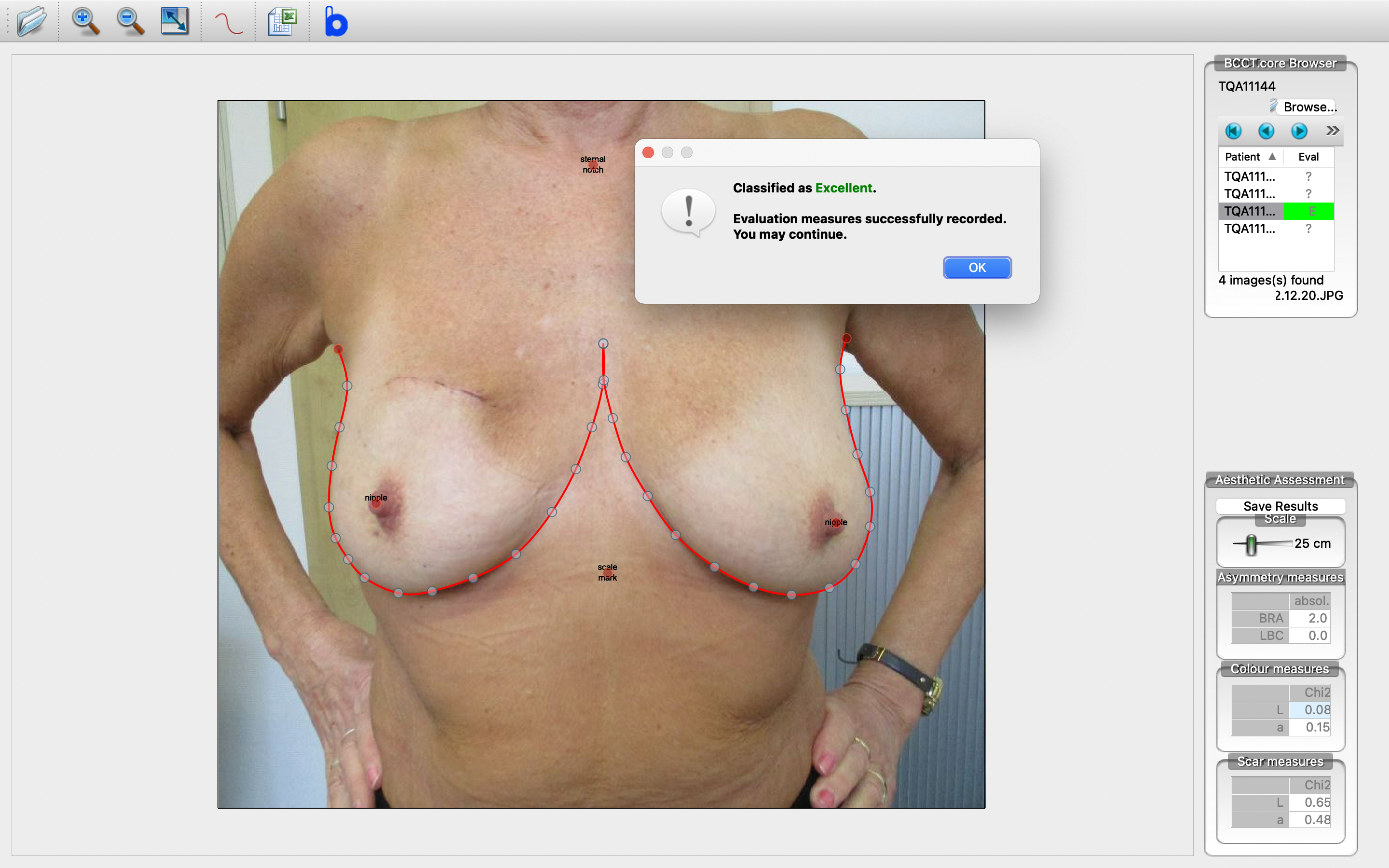 | Patient with excellent evaluation |
| --- | --- |
| 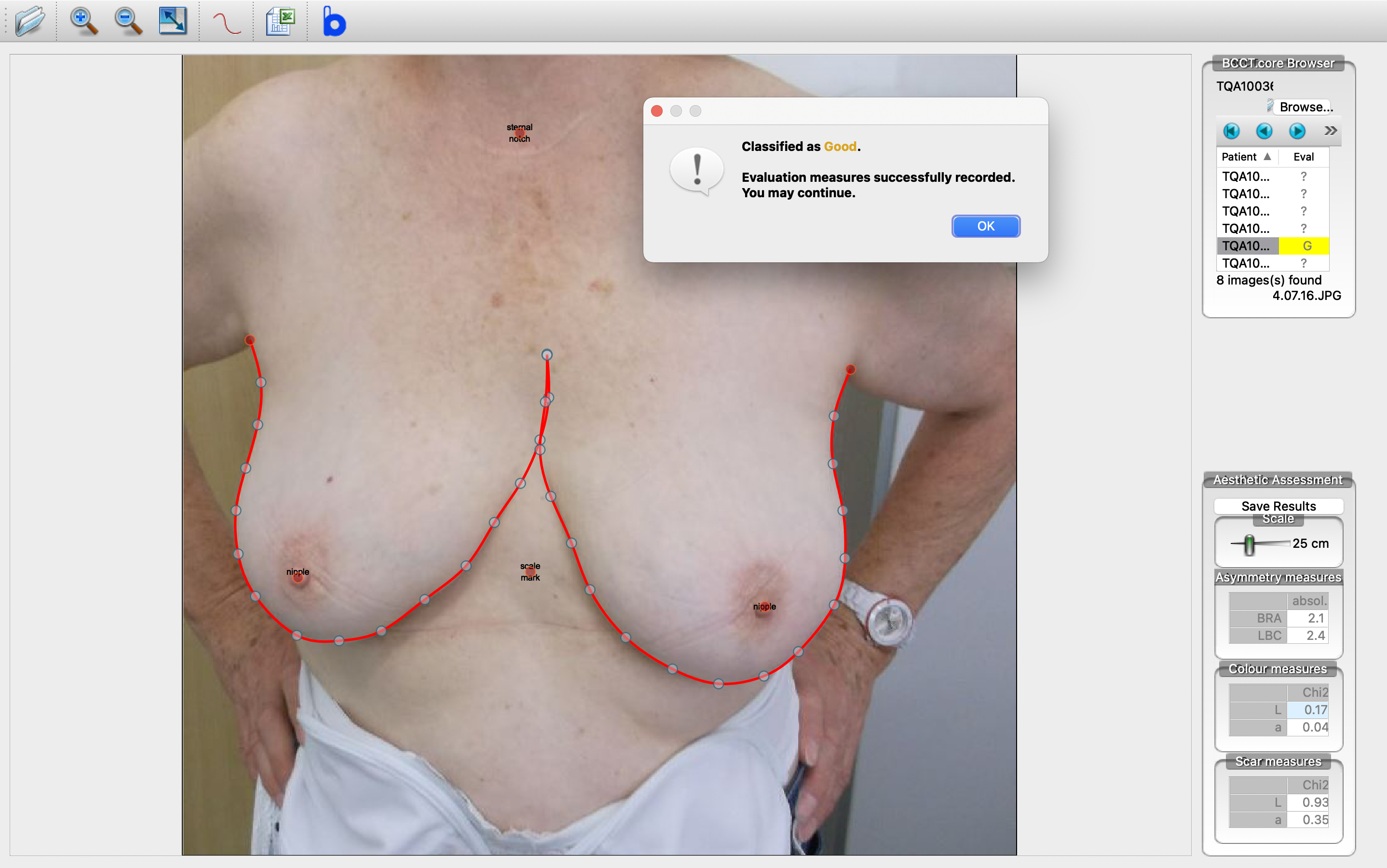 | Patient with good evaluation |
| 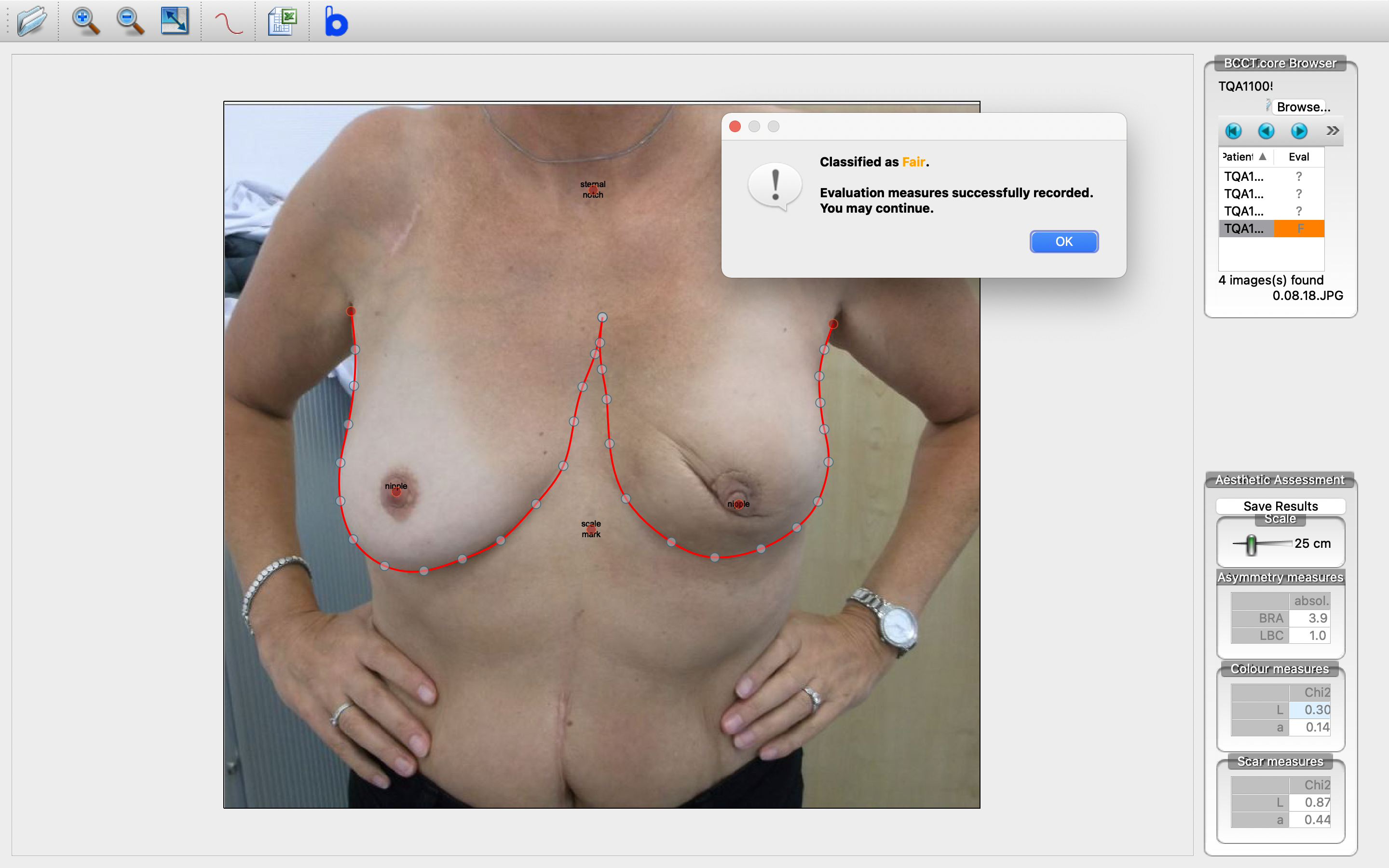 | Patient with fair evaluation |
| 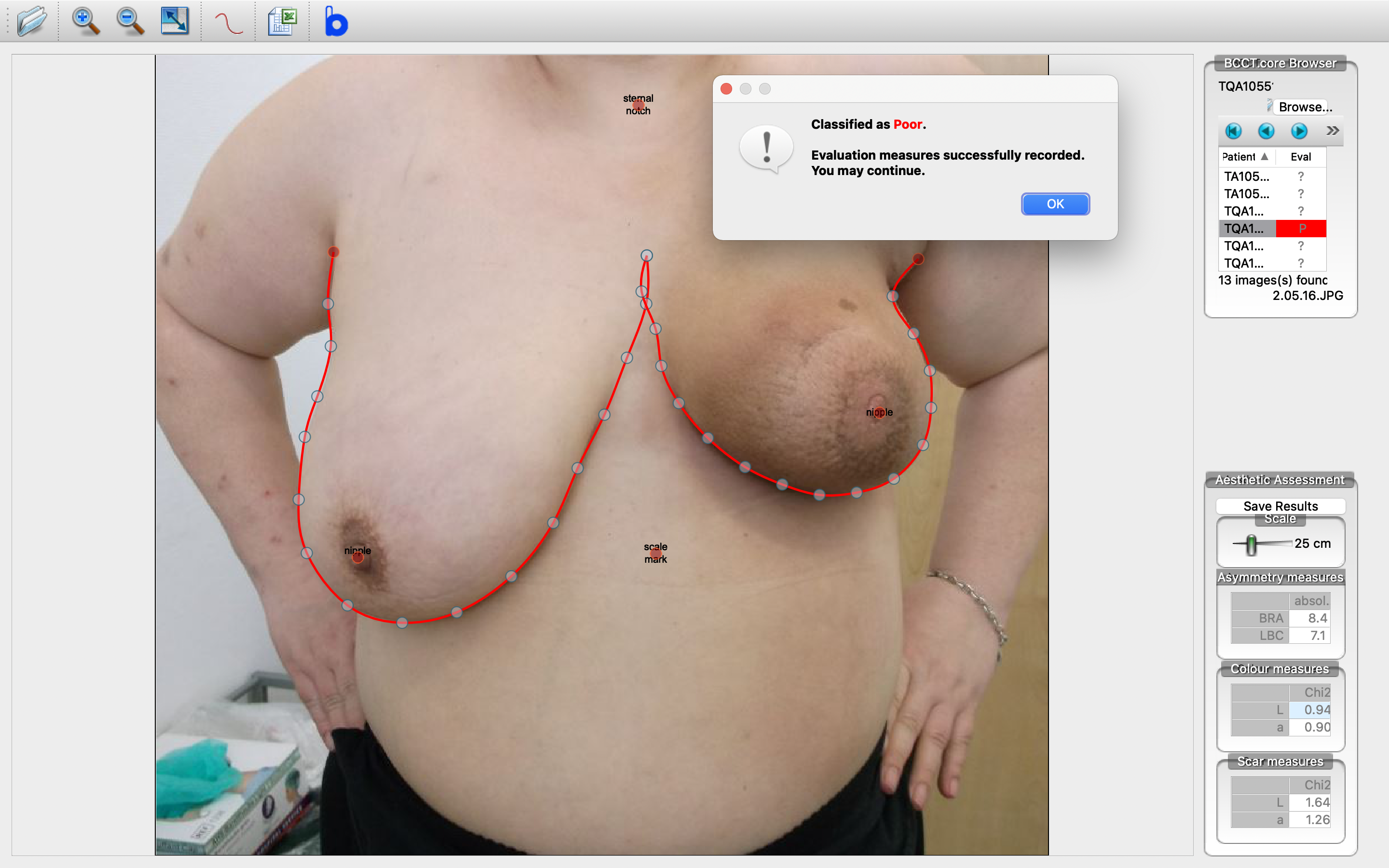  Supplement Fig. 1: Examples for BCCT.core categories from excellent to poor | Patient with poor evaluation |
